# Harnessing the Ion Transportome to Treat Cancer

**DOI:** 10.1113/JP290738

**Published:** 2026-10-18

**Authors:** Alina L. Capatina, Jodie R. Malcolm, Aamir Ahmed, Jason Bruce, Paul J. Buchanan, Thomas R. Eykyn, Elizabeth M. Fitzgerald, Amanda J. Foust, Tim G. Hales, Yoshikatsu Hayashi, Stuart G. Higgins, Alson Huang, Antony P. Jackson, Andrew D. James, Frederick Jones, Sarah J. Lilley, Karen McCloskey, Sara Moodad, Hugh Robinson, Samantha C. Salvage, Orkun S. Soyer, Serife Yerlikaya, Jonathan Ashmore, Ruth Murrell-Lagnado, William J. Brackenbury

**Affiliations:** 1 Jack Birch Cancer Research Unit, York Biomedical Research Institute, Department of Biology, University of York, YO10 5DD, UK; 2 Centre for Cell and Molecular Dynamics, University College London, WC1E 6JJ, UK; 3 Division of Cancer Sciences, School of Medical Sciences, Faculty of Biology, Medicine and Health, University of Manchester, M13 9PT, UK; 4 School of Nursing, Psychotherapy & Community Health, Dublin City University, D09 Y8VX, Ireland; 5 School of Biomedical Engineering & Imaging Sciences, King’s College London, SE1 7EH, UK; 6 School of Biological Sciences, Faculty of Biology, Medicine and Health, University of Manchester, M13 9PT, UK; 7 Department of Bioengineering, Imperial College London, SW7 2AZ, UK; 8 Institute of Academic Anaesthesia, University of Dundee, DD1 9SY, UK; 9 Department of Biomedical Sciences & Biomedical Engineering, University of Reading, RG6 7BE, UK; 10 School of Physics, Engineering & Technology & York Biomedical Research Institute, University of York, YO10 5DD, UK; 11 Department of Biochemistry, University of Cambridge, CB2 1QW, UK; 12 Metrion Biosciences Ltd, Cambridge, CB21 6AL, UK; 13 Department of Life Sciences, Manchester Metropolitan University, M15 6BX, UK; 14 Sophion Bioscience, Sussex Drug Discovery Centre, University of Sussex, BN1 9QJ, UK; 15 Johnston Cancer Research Centre, Queen’s University Belfast, BT9 7AE, UK; 16 Celex Oncology Ltd, London, W1W 6XH, UK; 17 Department of Physiology, Development and Neuroscience, University of Cambridge, CB2 3EL, UK; 18 Department of Molecular and Biomedical Sciences, City St George’s University of London, SW17 0RE; 19 School of Life Sciences, University of Warwick, CV4 7AL, UK; 20 Research Institute for Health Sciences & Technologies, Istanbul Medipol University, 34810, Turkey; 21 Department of Neuroscience, Physiology & Pharmacology, University College London, WC1E 6BT, UK; 22 School of Life Sciences, University of Sussex, BN1 9QJ, UK

**Keywords:** Ion channels, hallmarks of cancer, therapeutic resistance, cancer channelopathies, drug repurposing, technical development, ionic tumour microenvironment

## Abstract

Despite significant advances in early detection and therapeutic opportunities, cancer remains a critical global health challenge. The greatest barrier to reducing cancer-associated mortality is the persistent problem of therapeutic resistance and metastasis. Standard treatment approaches, including surgery combined with chemotherapy and radiation, are effective for many patients with localised disease. However, a substantial proportion of patients will experience recurrence and therapy resistance, leading to treatment failure and poor outcomes. This adverse prognosis for patients highlights the critical need to identify new therapeutic targets capable of improving long-term survival. The ion transportome comprises ion channels, solute transporters and pumps that regulate controlled movement of sodium (Na^+^), potassium (K^+^), calcium (Ca^2+^), chloride (Cl^-^), and hydrogen (H^+^) across cell membranes. Ion transport is central to many cellular processes, including cell growth, volume regulation, proliferation and migration. In many solid tumours, the finely-tuned ionic balance becomes dysregulated, with aberrant expression or activity of membrane transport proteins being closely linked to many of the hallmarks of cancer. Ion channel modulation using pharmacological agents designed to treat channelopathies has demonstrated promise in suppressing cancer progression in pre-clinical models. Thus, targeting ion-driven vulnerabilities within solid tumours may complement existing therapies to overcome resistance and improve patient outcomes. The dynamic nature of ion transport mechanisms means addressing critical research questions around their suitability as pharmacological targets is challenging, highlighting the need to develop innovative approaches to study these processes in oncology.

**Figure:**
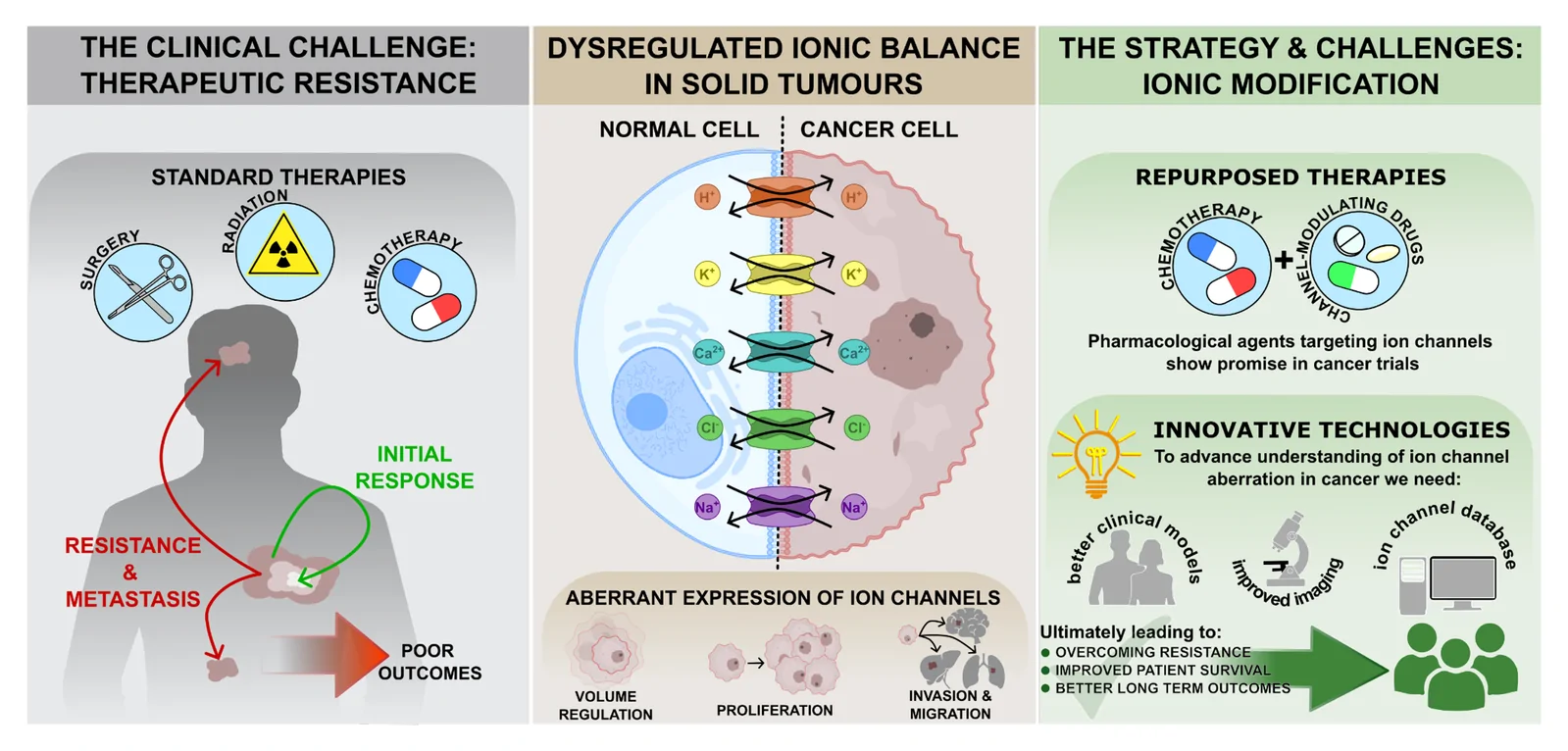
Despite improved treatment opportunities, a major challenge remains for a proportion of cancer patients whose tumours develop resistance to such therapies. These patients experience a poor prognosis, highlighting an urgent need for novel clinical targets to improve responses. In healthy cells, ion transport is central to many functions, and is tightly regulated by corresponding ion channels, transporters and pumps. In cancer cells, the ionic balance is dysregulated due to aberrant expression or function of these transport mechanisms, and has been implicated in pro-tumorigenic behaviours. Targeting ion channels and transporters in tumours may therefore be a viable therapeutic avenue. However, further work is required to fully explore the clinical potential of this class of targets. Improved models, technologies and databases will advance our understanding of ion channels/transporters and their contributions to malignancy.

Despite improved treatment opportunities, a major challenge remains for a proportion of cancer patients whose tumours develop resistance to such therapies. These patients experience a poor prognosis, highlighting an urgent need for novel clinical targets to improve responses. In healthy cells, ion transport is central to many functions, and is tightly regulated by corresponding ion channels, transporters and pumps. In cancer cells, the ionic balance is dysregulated due to aberrant expression or function of these transport mechanisms, and has been implicated in pro-tumorigenic behaviours. Targeting ion channels and transporters in tumours may therefore be a viable therapeutic avenue. However, further work is required to fully explore the clinical potential of this class of targets. Improved models, technologies and databases will advance our understanding of ion channels/transporters and their contributions to malignancy.

## Context and knowledge gap

The ion transportome is the complete assembly of proteins within a cell that function as membrane transporters, channels, carriers and pumps. These specialised proteins are dedicated to the movement of ions and other solutes across membranes to maintain cellular homeostasis, regulate signal transduction and generate electrochemical gradients. The role of ion channels in oncology was first recognised in the 1980-1990s (Binggeli and Weinstein, 1986). Since then, it has been shown that dysregulated expression, splicing and/or function of ion channels and transporters occur in many cancers (Pedersen and Stock, 2013). Altered ion channel and transporter activities in cancers can occur as a result of the repression of whole functional families, and/or selective upregulation of specific channel subtypes (Lang and Stournaras, 2014; Visentin et al., 2026). Oncogenic roles of ion channels have been identified in key cancer hallmarks, including abnormal proliferation, cell cycle regulation, angiogenesis, migration, invasion, and metabolic reprogramming (Cone and Tongier, 1971; Prevarskaya et al., 2018). More recently, there has been growing interest in organelle-expressed ion channels as oncological targets due to roles in metabolism, motility and autophagy regulation (Szabo et al., 2021). Additional studies have identified roles in regulating immune evasion (Erdogan et al., 2023) and cancer stemness (Samanta et al., 2025). Given the extensive evidence that ion channels and transporters exhibit recurrent dysregulation and specific alterations across malignancies, these observations provide strong rationale for considering the ion transportome as a clinically relevant landscape for cancer therapeutic intervention. However, the dynamic and transient nature of ion transport, and the relatively low expression of ion channels in cancer cells makes studying the dysregulated ionome challenging. Thus, development of assays with higher sensitivity and better spatiotemporal resolution is required for screening ion channels as novel biomarkers and therapeutic targets.

## Ionic homeostasis in normal and malignant cell cycle progression

**Figure 1:**
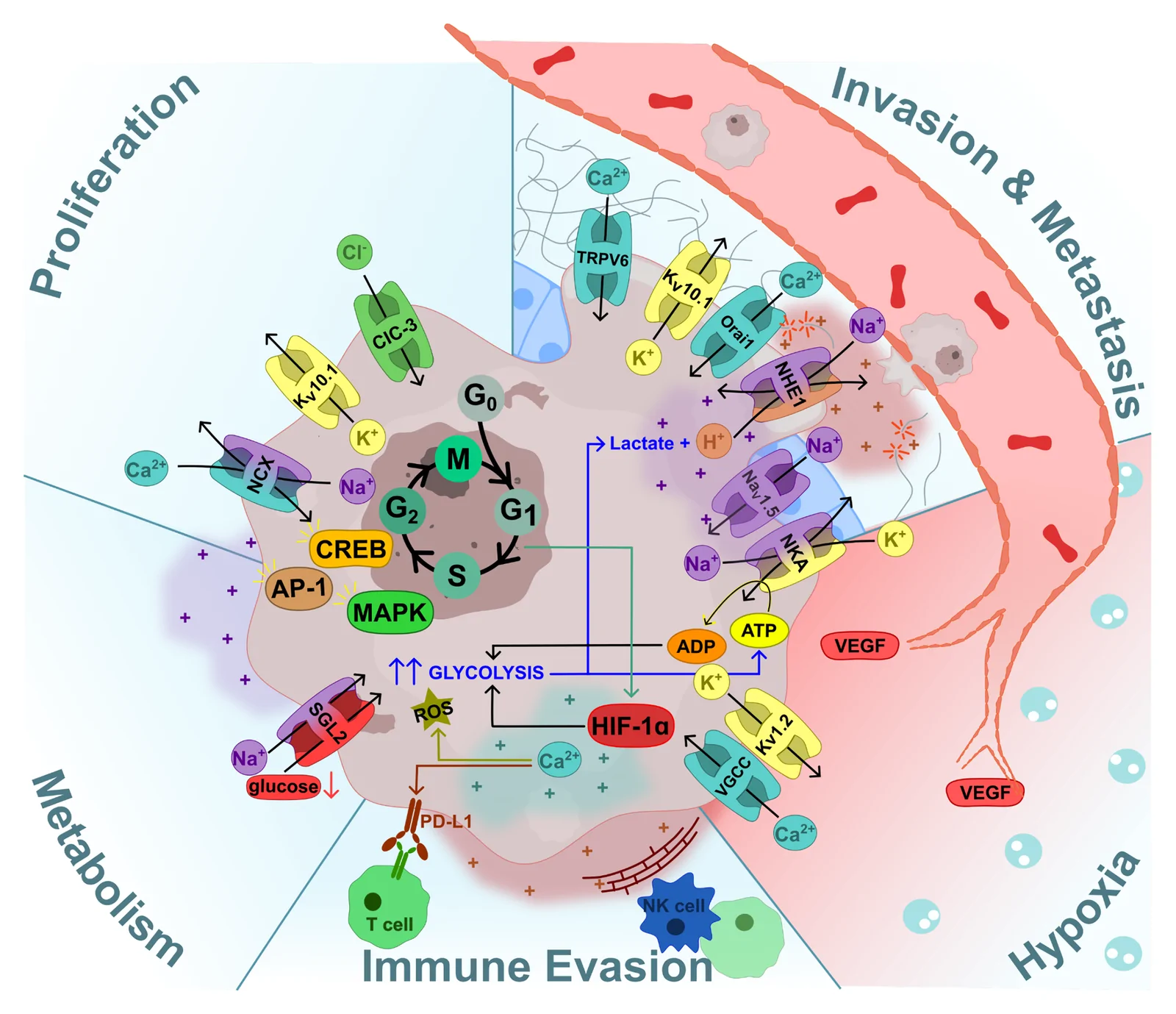
Ion channel dysregulation across key cancer processes. A schematic summary of ion channels implicated in core tumour behaviours. Highly proliferating cancer cells are observed to have a more depolarised cell membrane compared to quiescent cells. Ca^2+^ and Na^+^ dynamics are linked to progression through the cell cycle, being regulated through the NCX. K_v_10.1 has been implicated in cell cycle transition, particularly moving out of G_1_, whereas CLC-3 has a role in modulating M phase through regulation of cell volume. Altered ionic handling is critical for providing the capacity of malignant cells to invade surrounding tissue and metastasise to distant sites. Ca^2+^ influx through TRPV6 is linked to lymph node invasion and bone metastasis. K_v_10.1 exports K^+^ which further drives Ca^2+^ into the cell through Orai1, a process also tied to invasion and metastasis. Na^+^ import through Na_V_1.5 is associated with increased NKA activity in malignant cells, driving glycolysis and proton production, leading to extracellular acidification and digestion of surrounding ECM, as well as invadopodia formation, cytoskeletal remodelling, and activation of oncogenic signalling pathways. Aerobic glycolysis is a fundamental metabolic adaptation for cancer cell survival. To meet the heightened glucose demand of the cell, cotransporters such as SGLT2 are upregulated, enabling simultaneous import of glucose and Na^+^, further unbalancing ionic homeostasis. Elevated Na^+^ import increases NKA activity, requiring more ATP, resulting in a positive feedback loop sustaining glycolytic metabolism. Hypoxia and the activity of HIF-1α also feed into maintaining glycolysis, and induce formation of new blood vessels to support metastasis. Ca^2+^ and K^+^ signalling have been found to support the activity of HIFs in hypoxic cells. For cancer cells to escape immune surveillance, upregulation of PD-L1 enables scouting T cells to recognise cancer cells as “self” by complementary interaction with the PD-1 receptor on the immune cell surface. Upregulation of PD-L1 has been tied to elevated intracellular Ca^2+^ levels following SERCA inhibition. Furthermore, extracellular acidification functions as a barrier to infiltrating anti-tumorigenic NK and T cells, instead facilitating recruitment of pro-tumorigenic immune cells.

Membrane potential (V_m_) has been linked with proliferation and mitogenicity (Binggeli and Weinstein, 1986). It is widely understood that changes in V_m_ are correlated with transitions between G_1_/S and G_2_/M; hyperpolarised V_m_ is associated with quiescent somatic cells e.g., myocytes (Sundelacruz et al., 2009), whereas mitogenic or cancerous cells have more depolarised V_m_ (Figure 1). In addition, Na^+^ influx has been linked to rejoining of DNA strand breaks in lymphocytes, allowing rapid proliferation, although the mechanism is unknown (Prasad et al., 1987). Patch clamping, electrode arrays, and imaging recently revealed that V_m_ fluctuates on millisecond to minute time-scales in small-cell lung cancer (Peinado et al., 2025), glioma (Venkataramani et al., 2019), prostate cancer (Cabello et al., 2019), and several breast cancer cell lines (Quicke et al., 2022). These observations highlight that the link between V_m_ and cancer survival/progression might be more complex and expand beyond cell cycle regulation. Indeed, increasing evidence suggests that various Na^+^ transport mechanisms, including voltage-gated Na^+^ channels (VGSCs), play multiple roles in several aspects of cancer progression (Diaz et al., 2007; Shan et al., 2014; Lastraioli et al., 2021).

Ca^2+^ also plays a key role in cell cycle progression. Upon exiting G_0_, cells require Ca^2+^/calmodulin (CaM) signalling, while in early G_1_, Ca^2+^ promotes cell division by activating AP-1 and CREB, both transcriptional regulators of cell cycle proteins such as D-type cyclins. In cancer, Ca^2+^ dynamics are significantly altered compared to healthy tissue, often due to changes in the expression or function of key transporters. One example is the electrogenic Na^+^/Ca^2+^ exchanger (NCX), which transports Ca^2+^ out of (forward mode) or into the cell (reverse mode) in exchange for Na^+^ in the opposite direction. In cancer cells, NCX has been shown to function predominantly in reverse mode, in response to elevated intracellular Na^+^ concentration or membrane depolarisation (Brandalise et al., 2023), increasing cytosolic Ca^2+^. For example, in hepatocellular carcinoma (HCC) cells, TGF-β-mediated TRPC6 activation causes Na^+^ influx and membrane depolarization, leading to reverse mode operation of NCX1. These crucial transport events mediate the effects of TGF-β on the progression and metastasis of HCC (Xu et al., 2018). It has been proposed that NCX activity is transcriptionally regulated by hypoxic signalling, and promoted by acidic pH, which has been shown to inhibit forward mode NCX, impacting downstream Ca^2+^ signalling events (Hudecova et al., 2011; Leslie et al., 2019). T-type voltage-gated Ca^2+^ channels (VGCC) are also key players in cancer progression, bypassing the normal physiological Ca^2+^ transport/signalling mechanisms in non-excitable cells to trigger enhanced proliferation. Elevated expression of these channels is observed during the G_1_ and S phases of the cell cycle, primarily in oestrogen receptor-positive breast cancers, and increased confluency and physical contact amongst cells has also been shown to abolish currents associated with these channels (Panner and Wurster, 2006; Taylor et al., 2008).

Ca^2+^ also regulates mitogenesis by activating MAPK. Downregulation of the KCNN4 Ca^2+^-dependent K^+^ channel has been shown to induce both MAPK/ERK1/2 signalling and expression of matrix metalloprotease 9 in hepatocellular carcinoma cells (Li et al., 2020). Conversely, increased expression of the VGCC subunit CACNA2D3 is observed to inhibit tumour progression, induce apoptosis, and antagonise Wnt/cMyc-mediated proliferation (Wong et al., 2013; Wu et al., 2021). Voltage-gated K^+^ channels (VGKCs) have also been associated with hypoxia adaptations in cancer. K_v_10.1 has been shown to promote G_1_ progression and induce hypoxia-inducible factor (HIF)-1α activation, promoting angiogenesis (Ouadid-Ahidouch et al., 2001; Downie et al., 2008; Pardo and Stuhmer, 2014). In addition, the Cl^-^ channel ClC-3 regulates cell cycle transition by controlling cell volume through upregulation of Cl^-^ currents during M phase, and modulating the expression of Cyclin D1 (Ye et al., 2016). Interestingly, the regulation of ClC-3 channels seems to be modulated via Ca^2+^/CaM signalling, with the ClC-3 channel being in direct physical contact with the calmodulin-dependent protein kinase II (Cuddapah and Sontheimer, 2010).

The exact mechanisms by which ion channels and transporters become dysregulated in cancers are diverse, and include gene mutations and epigenetic changes that alter transcription, preferential selection of alternative splice variants, post-translational modifications, membrane trafficking of subunits, and mutations in tumour-suppressor pathways that regulate expression (Prevarskaya et al., 2018; Sun et al., 2020). Dysregulated ion homeostasis, via the aberrant expression of various ion channels and transporters, plays an important role in regulating cell cycle progression in tumour cells, presenting a unique, yet underexplored, therapeutic locus.

## Impact of ion transport on cellular physiology in cancer

### Invasion and metastasis

Growing evidence implicates several ion channels in the promotion of tumour invasion and metastasis (Figure 1). For example, in prostate cancer cells, expression of TRPV6 channels mediates Ca^2+^ entry and is linked to higher Gleason grading, increased proliferation, lymph node invasion and bone metastasis (Raphael et al., 2014). TRPV6 is similarly implicated in pancreatic ductal adenocarcinoma, with expression of this channel correlating to tumour stage, grade and cell proliferation (Mesquita et al., 2023). Local Ca^2+^ pulses coordinate lamellipodia retraction and focal adhesion turnover, promoting forward movement along the leading edge of migrating cells (Tsai and Meyer, 2012). In breast cancer cells, the VGSC Na_V_1.5 promotes invasion through upregulating the Na^+^/K^+^ ATPase (NKA) and promoting extracellular acidification, which facilitates extracellular matrix (ECM) degradation (Leslie et al., 2024). VGSC activity has also been linked to downstream signalling pathways regulating cytoskeletal dynamics and invasion across multiple cancer types (Brisson et al., 2013; Driffort et al., 2014; Guzel et al., 2019; Sanchez-Sandoval et al., 2023). In invasive ductal carcinoma, increased activity of K_v_10.1 hyperpolarises V_m_, which drives Ca^2+^ entry through Orai1, leading to enhanced migratory capacity, and has been linked to metastasis. Pharmacological blockade of this K^+^ channel with astemizole inhibits the metastatic potential of these cells *in vitro*, suggesting K^+^ efflux, and consequent V_m_ hyperpolarisation, are necessary for Ca^2+^-mediated invasion (Hammadi et al., 2012). Taken together, these findings demonstrate how aberrant ion channel activity regulates metastatic competence of malignant cells, enabling them to invade surrounding tissues and migrate to distant secondary sites, further positioning ion channels as compelling targets for therapeutic intervention.

### Metabolism

A major metabolic adaptation observed in many cancers is known as the Warburg effect (Racker, 1972). Intensively proliferating tumours often stabilise hypoxia-associated transcription factors (HIFs) such as HIF-1α to cope with the limited supply of O_2_. HIF-1α activates glycolytic enzymes and promotes aerobic glycolysis as the major energy-producing mechanism in the cell, increasing the requirement for glucose, which is thought to be connected with Na^+^ dynamics (Counillon et al., 2016; Jeong et al., 2021). One connection comes from the Na^+^-glucose co-transporter 2 (SGLT2) that is frequently upregulated in cancer and facilitates the concomitant import of glucose and Na^+^. This transport of glucose occurs even when extracellular glucose is low, as it is driven by the Na^+^ gradient. In agreement with this, tumoral Na^+^ is elevated compared to healthy tissues in various malignancies, including breast, prostate, and pancreatic cancers (Dutka et al., 2022).

Increased Na^+^ import increases NKA activity, elevating ATP demand. NKA primarily uses glycolytic ATP, leading to heightened need for glycolysis, creating a positive feedback loop, maintaining glucose uptake and perpetuating Na^+^ import (Michaels et al., 2024). Treatment with the NKA inhibitor ouabain elevates intracellular Na^+^ and inhibits glycolytic flux in triple-negative breast cancer (TNBC) cells. Opening VGSCs using veratridine increases ATP usage, presumably by augmenting NKA activity; while inhibition of VGSCs using tetrodotoxin decreases extracellular acidification rate, suggesting a drop in glycolytic activity (Leslie et al., 2024).

Live-cell measurements also showed a linear correlation between NKA activity and glycolytic flux. This was validated *in vivo* in TNBC xenografts, where treatment with ouabain significantly decreased glycolytic flux measured as ^18^F-FDG uptake (Michaels et al., 2024), and MRI studies showed that elevated intracellular Na^+^ correlates with tumour progression (James et al., 2022). Together, these observations highlight altered ionic balance as a key determinant of cancer cell metabolism, further suggesting Na^+^ transport modulation as a novel therapeutic opportunity.

### Hypoxia

Hypoxia strongly influences the ion transportome in several types of malignancy. In breast cancer cells, low O_2_ induces expression of K_v_10.1 and enhances the current through these channels (Laîné et al., 2025), promoting hypoxia-mediated cell migration by inducing a mesenchymal phenotype. Furthermore, knockdown of K_v_10.1 reduces HIF-1α protein levels, implicating this channel in the hypoxic response through a positive feedback loop (Laîné et al., 2025). In osteosarcoma tissues, HIF-1α binds to the promoter of the inwardly rectifying K^+^ channel, KCNJ2, to increase its expression under hypoxic conditions. Interestingly, HIF-1α also binds directly to KCNJ2, inhibiting its degradation, to establish a positive feedback loop whereby hypoxia induces the expression of K^+^ channels, which then act to sustain the hypoxic transcriptional response (Shen et al., 2023).

Ca^2+^ signalling also contributes to stabilisation and activation of HIF-1α, through calmodulin kinase–dependent ERK phosphorylation that promotes HIF-1α nuclear accumulation, and phosphorylation of co-factor p300 (Mottet et al., 2003; Shimoda and Polak, 2011). In hypoxic glioma cells, TRPC6 is activated, leading to elevated intracellular Ca^2+^ levels. This increased Ca^2+^ influx inhibits the activity of PHD, promoting the stabilisation of HIFs (Li et al., 2015). In addition, specific Ca^2+^ channels can directly modulate hypoxia signalling. For example, TRPC1 has been shown to regulate HIF-1α translation and promote epithelial-mesenchymal transition (EMT) via Snail and Claudin-4 activation (Azimi et al., 2017; Wu et al., 2021). Moreover, Ca_v_1.3 modulates HIF-1α signalling under hypoxia, influencing tumour behaviour during androgen deprivation in prostate cancer (O’Reilly and Buchanan, 2022).

Cl^-^ channels also contribute to the hypoxic response in solid tumours. In pancreatic cancer, ECM stiffness activates the Wnt/β-catenin/TCF4 signalling pathway, leading to upregulation of the Cl^-^ intracellular channel (CLIC1). Elevated CLIC1 expression correlates with glycolysis, linking this channel to metabolic reprogramming associated with the Warburg effect. Mechanistically, CLIC1-driven metabolic shifts impair prolyl hydroxylation in a reactive oxygen species (ROS)-dependent manner, thereby stabilising HIF-1α. These findings suggest that targeting the CLIC1/ROS/HIF axis to inhibit aerobic glycolysis may disrupt HIF-dependent tumour progression (Zheng et al., 2024). Similarly, CLIC1 regulates the MAPK/ERK pathway in colon cancer cells, promoting migration and invasion in a ROS-dependent manner during hypoxia-reoxygenation (Wang et al., 2014). In contrast, low expression of the Ca^2+^-activated Cl^-^ channel regulator (CLCA1) is associated with poor survival in pancreatic cancer patients. Here, overexpression of CLCA1 impairs HIF-1α stabilisation by downregulating SIRT1 expression. In this case, activating CLCA1 could be a strategy for overcoming hypoxia-induced chemoresistance in pancreatic cancer patients (Hu et al., 2026).

Na^+^ transport is also connected to the hypoxic response in cancer. For example, in TNBC cells, hypoxia increases the expression of NHE1 in lamellipodia, and this cotransporter promotes EMT-dependent cell migration. Importantly, this hypoxia-driven NHE1-dependent motility can be ablated through use of PPARγ antagonists (Takatani-Nakase et al., 2022). Hypoxia also induces expression of Na^+^-driven bicarbonate transporters (NDBTs), *SLC4A4* and *SLC4A5*, in TNBC models. Enhanced expression of NDBTs supports metastasis and invasion of breast cancer cells by inducing EMT and altering metabolic activity to increase ATP and extracellular lactate levels (Carroll et al., 2022). VGSCs are also linked to hypoxic adaptation. In breast cancer, prostate cancer, and melanoma models, hypoxia enhances persistent inward Na^+^ current carried by VGSCs and confers increased sensitivity to the VGSC inhibitor ranolazine (Djamgoz, 2024). Treatment of hypoxic TNBC cells with the β-adrenergic receptor (β-AR) antagonist propranolol in combination with ranolazine, suppresses cell motility and invasion. In addition, propranolol alone decreases total neonatal Na_V_1.5 (nNa_V_1.5) protein expression, the predominant VGSC isoform expressed in these breast cancer cells, thereby diminishing the Na^+^ channel signalling that supports invasive behaviour (Guzel et al., 2019; Lee et al., 2019). Together, these studies highlight VGSCs such as nNa_V_1.5 as key mediators of hypoxia-driven metastatic behaviour.

### Immune evasion

Ion transporter activity has been implicated in mechanisms leading to immune evasion. For example, Ca^2+^ signalling enhances mitochondrial ROS production in solid tumours, activating the immunomodulatory and tumorigenic transcription factor STAT3 (Gong et al., 2001). Interestingly, glutamine deprivation inhibits the sarco/endoplasmic reticulum Ca^2+^ ATPase (SERCA), leading to activation of Ca^2+^-mediated NF-kB signalling, upregulating the PD-L1 immune checkpoint, thus enhancing immune evasion (Byun et al., 2020; Wu et al., 2021). In glioma, high levels of ions and metabolites secreted into the TME, such as K^+^, limit the function of infiltrating T cells (Hansen and Jackson, 2025). NKA activity in TNBC and lung cancer cells enhances expression of the IDO1 immune checkpoint, potentially facilitating immune evasion (Shandell et al., 2022). Furthermore, acidification of the tumour microenvironment from increased NKA-driven glycolytic reliance and export of H^+^ also functions as a tumour-protective barrier, inducing anergy in tumour-infiltrating cytotoxic T and NK cells, while facilitating recruitment of regulatory T and myeloid-derived suppressor cells, which neutralise anti-tumour immunity (Cappellesso et al., 2024; Liu et al., 2024). Accordingly, drugs targeting lactate metabolism are becoming a key component of therapy in the immuno-oncology field (Rahman et al., 2024). In patients with head and neck squamous cell carcinoma (HNSCC), those who had positive outcomes to the immunotherapy pembrolizumab had increased KCNN4 activity in tumour infiltrating lymphocytes (TILs) immediately after treatment, accompanied by an increase in K_V_1.3 activity and Ca^2+^ fluxes, compared to those for whom immunotherapy was not successful. Enhanced K^+^ and Ca^2+^ fluxes in TILs are associated with improved chemotaxis of CD8+ T cells, enabling them to navigate TME more effectively. These electrophysiological changes highlight how modulating ion transport can elicit beneficial therapeutic effects to overcome immune evasion and promote immune infiltration (Newton et al., 2020).

### Cancer-associated fibroblasts

Cancer-associated fibroblasts (CAFs) are the most abundant non-malignant cell type within the stroma of solid malignancies. CAFs structurally alter the ECM and engage with immune cells to promote an immunosuppressive TME, enhancing therapy resistance (Luo et al., 2025). Altered ion channel expression in CAFs further reinforces tumour growth, progression, and immune evasion. Activated CAFs reportedly display increased K^+^, Na^+^ and Ca^2+^ conductance, enhancing contractility and ECM remodelling (Huang et al., 2025). For example, in pancreatic ductal carcinoma (PDAC), pancreatic stellate cells, which are considered the main precursors of CAFs, upregulate Orai1, which promotes proliferation and the development of a myofibroblastic phenotype in CAFs (Schleinhege et al., 2025). Similarly, TRPM7 is also overexpressed in CAFs, and is associated with pancreatic stellate cell activation, where it regulates proliferation through modulation of cell cycle regulators such as p53 via the PI3K/Akt pathway (Auwercx et al., 2022). Inhibition of the NKA with cardiac glycosides, e.g. digoxin, blocks TGF-β-induced remodelling of the ECM across multiple fibroblast cell lines. This inhibition of CAF induction is due to reduced SMAD promoter activity downstream of TGF-β signalling (Coleman et al., 2016).

### Cell death

As well as being essential for sustaining cancer cell viability, ion channels and transporters play critical roles in regulating programmed cell death. Ion-dependent cell death pathways are often hijacked by tumour cells to sustain their survival, enabling them to bypass signalling checkpoints that would otherwise commit to apoptosis. For example, in cervical and ovarian cancer cell lines, blockade of K_Ca_1.1 channels triggers apoptosis, characterised by increased expression of p53, p21 and Bax, alongside decreased levels of hsp70 and hsp90, linking BK channel activity to sustaining an anti-apoptotic phenotype (Han et al., 2007). Conversely, in TNBC cells, K_Ca_1.1 activation induced apoptosis in a mechanism associated with mitochondrial membrane depolarisation and increased intracellular Ca^2+^ (Ma et al., 2012). In oesophageal squamous cell carcinoma, K_v_11.1 activity inhibits apoptosis by stimulating the PI3K/Akt pathway, promoting proliferation and inducing EMT. Knockdown of this K^+^ channel reduces tumour growth and metastasis in vivo (Wang et al., 2019).

NCX1 is upregulated in several tumour types, and in gastric cancer its elevated expression is associated with poor clinical outcomes. As a key regulator of Na^+^ and Ca^2+^ homeostasis, NCX1 prevents intracellular Ca^2+^ overload that can trigger Ca^2+^-dependent cell death. Pharmacological blockade of forward-mode NCX1 using CB-DMB triggers death of gastric cancer cells by inhibiting Ca^2+^ efflux and thus contributing to lethal Ca^2+^ accumulation (Laurino et al., 2025). CB-DMB has also been explored for its pro-apoptotic potential in glioblastoma and melanoma (Hu et al., 2019; Liu et al., 2022), highlighting how shifts in Ca^2+^ flux can have significant consequences for cancer cell survival. In HNSCC cells, overexpression of the TMEM16A Ca^2+^-activated Cl^-^ channel correlates with increased tumour size, decreased pro-apoptotic Bim protein levels, reduced apoptotic activity and a suppressed response to cisplatin. Knockdown of TMEM16A restored chemotherapy sensitivity, evidenced by increased apoptotic cell death (Godse et al., 2017).

Taken together, these processes illustrate how dysregulated ionic homeostasis is connected across key cancer mechanisms which reinforce one another and enable malignant cells to sustain growth, adapt to stress, avoid apoptosis, and remodel their microenvironment (Figure 1).

## Recent advances in ion transport-targeting anti-cancer therapies

### Repurposing ion transport-targeted drugs

There is a growing body of evidence supporting the repurposing of existing ion channel/transporter-modulating therapeutics for use in the treatment of cancers. Table 1 provides a representative (non-exhaustive) selection of ion transport-targeting compounds that have been reported to influence tumour behaviour. For example, the VGSC-inhibiting anti-epileptic phenytoin and anti-anginal ranolazine reduce breast cancer invasiveness and lung colonisation *in vivo* (Driffort et al., 2014; Nelson et al., 2015). In colorectal carcinoma patients, the amyotrophic lateral sclerosis therapeutic, riluzole, improves response to the FOLFOX treatment regime where resistance had previously occurred (Li et al., 2025). In addition, the local anaesthetic lidocaine inhibits Na_v_1.5-mediated EMT, reducing migration of ovarian cancer cells (Liu et al., 2021), and suppresses metastasis in a murine breast cancer model (Freeman et al., 2019). Moreover, perioperative peritumoral lidocaine administration leads to improved disease-free and overall survival in breast cancer patients (Badwe et al., 2023). However, despite some promising observations in clinical studies surrounding the use of perioperative systemic lidocaine administration, it is important to recognise that in some instances lidocaine has failed to demonstrate significant improvements in survival, and has even demonstrated adverse effects in some cell models (Wall and Buggy, 2021). For example, although a high concentration of lidocaine has no significant impact on colorectal cancer cell viability, low concentrations have been shown to enhance the viability of metastatic cell lines (Siekmann et al., 2019).

VGCC inhibitors indicated for hypertension (e.g., lacidipine, manidipine, benidipine, nifedipine) or migraines (lomerizine) reduce expression of cancer stem cell markers (e.g. OCT3/4, NANOG) in ovarian cancers (Lee et al., 2020), possibly by lowering intracellular Ca^2+^, thus disrupting mitochondrial metabolism and transcriptional programs that maintain stemness. Similarly, VGCC inhibitors reduce VGCC activity in prostate cancer cells under hormone therapy, suggesting repurposing potential (McKerr et al., 2023). However, incorporation of VGCC blockade into cancer therapy will require clinical validation, particularly for isoform-specific agonists and antagonists that may resolve the nuanced roles of individual channel subtypes (Bhargava and Saha, 2019). Further examples include anti-metastatic and anti-proliferative effects of memantine in acute lymphocytic leukaemia cells via inhibition of K_v_1.3 (Lowinus et al., 2019), and inhibition of K_v_10.1-mediated TNBC cell migration by chloroquine and astemizole (Valdés-Abadía et al., 2019). In addition, zafirlukast was shown to suppress the metastatic behaviour of lung cancer cells via inhibition of ANO1 channels (Shi et al., 2022). Together, these studies highlight the potential of ion channel-targeting strategies for limiting tumour progression across different cancer types. While many of the studies are promising, careful interpretation is necessary given that some channel modulators show minimal efficacy or adverse effects depending on cellular context and dosing.

### Novel ion transport-targeting therapies

An alternative approach has been to explore non-clinically indicated ion transport-modulating compounds for their anti-cancer effect. For example, several approaches to blocking TRP channel activity have been explored. The TRPC6 inhibitor BI-749327 prevents mammosphere formation in breast cancer stem cell cultures (Mukhopadhyay et al., 2023) and the TRPV1 antagonist AMG9810 reverses cisplatin resistance in cervical cancer cells (Oh et al., 2023). The TRPV6 inhibitor Soricidin-13 has demonstrated promise in advanced solid tumours, including pancreatic tumours, showing evidence of stable disease in patients who were refractory to all other standard-of-care treatments (Fu et al., 2017). Moreover, mAb82, which also targets TRPV6, significantly decreased prostate cancer cell survival and reduced tumour growth *in vivo* (Haustrate et al., 2024).

The small molecule T-type VGCC blocker KTt-45 was shown to induce apoptosis in various cancer cell lines (Du et al., 2023). In addition, targeting Ca^2+^ release-activated Ca^2+^ (CRAC) channels, inhibiting downstream store-operated Ca^2+^ (SOC) channels, has been proposed as an anticancer strategy (Stauderman, 2018). For example, the CRAC channel inhibitor RP4010 suppresses tumour cell proliferation and enhances the efficacy of chemotherapy in PDAC (Khan et al., 2020) and oesophageal squamous cell carcinoma models (Cui et al., 2018). However, the CRAC channel subunit Orai1 also has a pro-apoptotic function, thus, Orai1 inhibitors may lead to apoptosis resistance. On the other hand, Orai3 is also over-expressed in cancer cells but does not have a similar role in apoptosis. Therefore, targeting Orai3, rather than Orai1, might be a better therapeutic strategy (Dubois et al., 2014).

The novel food-derived compound, liensinine, was shown to be a highly potent K_v_10.1 inhibitor with anti-proliferative and anti-migratory effects in hepatocellular carcinoma models (Ma et al., 2024). Similarly, the natural compound procyanidin B1, which is extracted from the grape seed, was shown to exert anti-tumour effects in liver cancer cells *in vitro* and *in vivo* through selective targeting of the K_v_10.1 channel (Na et al., 2020) Interestingly, blocking Cl^-^ transport through Ca^2+^-activated ANO1 channels with the natural compound Schisandrathera has been shown to elicit antitumor effects in prostate and oral cancers (Park et al., 2023). Novel compounds to attenuate Na^+^ transport have also been explored. The spider venom peptide JZTX-14, which broadly inhibits VGSC activity, has been shown to impair EMT in TNBC cells (Zhang et al., 2018; Wu et al., 2023). The antibody E3Ab, which specifically targets Na_V_1.5, reduced ovarian tumour growth and cellular invasion (Gao et al., 2019). Inhibition of Na^+^ transport by blocking the Na^+^-glucose transporter type 2 (SGLT2) with dapagliflozin is also being investigated for its cardioprotective potential in breast cancer patients undergoing anthracycline-based treatment, with or without trastuzumab (Greco et al., 2025), highlighting interest in targeting the transportome for applications beyond attenuating tumour progression.

It is important to note that, while many clinical and experimental studies focus on inhibiting ion transport as a therapeutic strategy in cancer, there is also growing interest in the potential benefits of activating some ion transportome pathways. For example, activation of the Kir6/SUR channel complex with the anti-hypertensive agent, minoxidil, induces cell death in endometrial and ovarian cancer cells, resulting in reduced tumour growth (Fukushiro-Lopes et al., 2020). Similarly, activation of the cold-sensitive TRPM8 channel has been shown to exert anti-tumour effects in breast cancer cells through AMPK-dependent signalling (Huang et al., 2020), whereas in bladder cancer models activation of this channel with menthol induces cell death via mitochondria regulation (Li et al., 2009). These findings illustrate that both ion channel inhibition and activation may represent viable therapeutic approaches, depending on the biological context and the specific transport mechanisms involved.

In summary, several ion channel modulators have undergone evaluation across *in vitro* and *in vivo* models, as well as in early-stage clinical trials, for their potential to complement existing treatment for cancer patients (Table 1) (Zhou et al., 2025). Although some show promise, limitations come from broad target and/or tissue specificity, narrow therapeutic index and their inability to distinguish between cancerous and healthy cells.

## Technological challenges and advances in studying the ionic tumour microenvironment

The rapid and transient nature of ionic movement makes it difficult to capture with conventional imaging approaches, e.g., confocal microscopy. Studying cancer-associated channelopathies is further hindered by limited isoform-specific antibodies and the inability of standard RNA-sequencing to distinguish subtle splice variants. While ion-sensitive probes exist, multiplexing is technically challenging. Manual patch clamping requires specialised expertise, and *in vivo* electrophysiology is invasive and relatively nonspecific. Collectively, these barriers prevent large-scale analysis of the ionic landscape in solid tumours. Overcoming these shortfalls requires innovative electrophysiology tools and scalable imaging modalities to provide robust pharmacodynamic markers for drug development and therapeutic discovery.

Disrupting ionic signalling between cancer cells is a desirable therapeutic strategy, despite the technical challenges described above. At the single-cell level, the advance of automated patch-clamping has brought substantial gains in recording throughput. High-throughput electrophysiology facilitates the comparative exploration of altered electrophysiological characteristics between healthy and malignant cells, cells originating from different types of tumour and between cells receptive and resistant to existing and novel therapies (Shi et al., 2025).

With the preclinical adoption of more complex 3D cell-based modelling systems such as spheroids and organoids, the capture of extracellular electrical activity in culture by microelectrode arrays (MEAs) has been limited by their 2D recording interface and the challenge of amplifying small extracellular voltages in non-electrogenic cell types. However, approaches such as 3D, flexible, and/or self-folding MEAs can improve cell-electrode coupling, enabling the recording of coordinated field potential changes in 3D cellular systems (Pesantez Torres et al., 2025). These could give valuable insight into ionic communication within 3D models of the tumour microenvironment and the effect of pharmacological perturbations.

Genetically encoded physiological sensors enable detection of dynamic ion concentrations or V_m_ within living cells (Zaver et al., 2023). The most abundant class of these probes enable Ca^2+^ imaging, e.g., jGCaMP8 Ca^2+^ sensors that have superior kinetics and specificity compared to earlier iterations of these probes, and thus enable tracking of Ca^2+^ spikes at exceptional temporal resolution in live cells and in animal models (Zhang et al., 2023). Specific development of voltage sensors has resulted in voltage-sensitive dyes (Fiala et al., 2025) and genetically encoded indicators that have sub-millisecond responses to V_m_ changes (Hao et al., 2024), offering researchers the ability to study dynamic voltage fluctuations with exceptional timing precision. Recently, ultra-sensitive sub-millivolt V_m_ indicators that enable imaging across the full physiological resting range have been developed, expanding capacity to understand how bioelectric signalling contributes to tumorigenesis (Rühl et al., 2026). Deeper penetration of tissue has also been made possible by the advent of red-shifted voltage-sensing dyes (Yan et al., 2023). Together, these reporters enable faster temporal resolution for capturing rapid ionic and V_m_ oscillations with high throughput across heterogeneous cell populations.

Correlative imaging techniques combining multiple microscopy approaches have significantly revolutionised the study of the ionic environment in cells and tissues. Scanning ion conductance microscopy, a technique used to map the topology and electrochemical impedance of living cells, is often used in combination with confocal microscopy and can be implemented with patch clamping to record electrical signals while imaging the cell surface in real time (Shi et al., 2018; Gong et al., 2023). Recently, mass spectrometry has propelled insights into the importance of elemental distribution across the tumour microenvironment: laser ablation-inductively coupled plasma-mass spectrometry with time-of-flight mass spectroscopy has enabled novel understanding on how ions are distributed across tumour samples from different stages of malignancy (Escudero-Cernuda et al., 2025). In the clinical setting, the ability to monitor Na^+^ concentrations in malignant tumours has attracted interest in recent years. ^23^Na-MRI has shown that intratumoral Na^+^ correlates with malignancy and is predictive of chemotherapy response in breast cancer patients (Zaric et al., 2021). The development of ultrahigh field scanners and specialised Na^+^ acquisition sequences further advances the possibility of imaging Na^+^ in other types of cancer (Poku et al., 2021). Together, the emergence of these imaging techniques has provided new avenues for interrogating ionic distribution and its involvement in malignancy.

## Remaining limitations and emerging opportunities for studying the ionic tumour microenvironment

Studying ion transport is challenging due to its involvement in every aspect of cell survival and homeostasis. However, there remain important gaps in knowledge and techniques that should be addressed to further catalyse strategies targeting cancer-driving channelopathies. For example, while mutations in tumour-suppressor pathways have been linked to altered ion channel and transporter gene expression in some cancers (Sun et al., 2020), further work is required to elucidate the mechanistic interactions between major cancer drivers (e.g., *TP53, PI3K*) and ion channel/transporter function, particularly how recurrent oncogenic mutations reshape cellular electrophysiology in malignancy.

Importantly, unless specific mutations differentiate cancer-promoting ion channels from healthy tissues, pharmacological target specificity could be difficult to achieve (Pardo and Stuhmer, 2014). As a result, channel-targeted therapies may produce off-target toxicities, including cardiotoxic, neurological, and muscular side effects. Nevertheless, identifying cancer-specific isoforms, splice variants, or tumour-restricted expression patterns should mitigate these challenges and preserve therapeutic potential. Furthermore, although many studies implicate various ion channels in tumour-promoting and pro-metastatic processes, ionic dysregulation is not the only source of malignancy. Thus, ion channel therapies may serve as adjuvants in combination with other treatments. Future research should focus on understanding how to combine ion transport modulators with existing anticancer therapies in order to create more effective and readily implementable treatment regimes.

A key limitation in advancing ion channel targeted therapies lies in the lack of preclinical models that faithfully recapitulate tumour bioelectrical states and their relationship to tumour heterogeneity. While animal models provide a valuable *in vivo* context, species-specific differences in ion channel expression, pharmacology, and electrophysiological behaviour can significantly impact translatability. For example, differential sensitivity of ion transporters, e.g. NKA, to pharmacological inhibitors, highlights the challenge of extrapolating findings across species (Akimova et al., 2015). More fundamentally, conventional models often fail to capture the dynamic and spatially regulated nature of ionic signalling within the tumour microenvironment. Ion fluxes are tightly coupled to local factors such as hypoxia, extracellular matrix composition, and cell–cell interactions, which are not adequately represented in standard *in vitro* or *in vivo* systems. While patient-derived xenografts and 3D culture models offer improvements, they remain limited in their ability to model immune interactions and the complex bioelectrical heterogeneity of human tumours (Jin et al., 2023). All these aspects limit our understanding of ion transport in human cancers and highlight the need for developing better model systems, potentially integrating with emerging technologies such as single-cell and spatial transcriptomics (Zhou et al., 2025).

From a technical perspective, there is a common interest amongst ion channel researchers in improving imaging modalities to better capture ionic and voltage dynamics across different scales, from simple 2D *in vitro* systems to *in vivo* model organisms and, ultimately in clinical settings. Of profound significance is the desire to image multiple ions and voltage concurrently with high temporal resolution using non-invasive and high-resolution techniques. Current technologies that enable elemental imaging *in vivo* are particularly lacking in spatial resolution, making accurate multi-modal alignment challenging. In addition, although the altered bioelectric state of cancer cells, including plasticity of metabolic/ionic gradients, is known to play a crucial role in driving tumour progression and invasiveness, these bioelectrical dynamics are poorly characterised due to the complex and multiscale feedbacks between biochemical and electrical processes. This key knowledge gap raises a need for multiscale mathematical models that integrate ion channel activity, membrane potential, and metabolic fluxes in cancer cells and tumour tissue (Cervera et al., 2014; Schofield et al., 2020).

Despite significant evidence surrounding the role of ion transport in cancer development, there remains an unmet need to establish a central resource to categorise cancer-associated channelopathies. There is an exciting opportunity to generate an “onco-channelopathy repository” where researchers could deposit electrophysiological, transcriptomic, proteomic or imaging datasets relevant to ion channel aberrations in malignancies into an open-access database specifically designed to better disseminate knowledge to the wider ion channel community. Recent efforts to consolidate membrane transport information include the newly developed Membrane Transport Protein Database (MTDB) (Visentin et al., 2026), which provides a resource for cataloguing the transportome and functional roles across physiological systems. Implementation of the MTDB has identified common ion channel genes that are dysregulated across multiple tumour types, highlighting the significant value of such a tool (Visentin et al., 2026). Hence, the MTDB lays important groundwork for the future development of an oncology-focused channelopathy repository in which electrophysiological readouts could complement omics datasets and link ion channel expression with functional bioelectrical phenotypes in tumours. Such a database could add to data on ion channel mutations from the Cancer Genome Atlas (Cancer Genome Atlas Research Network et al., 2013), organise malignant tumour types based on the dysregulated expression of specific ion transporters, retain relevant mutational signatures, and highlight “hot topics” of ion channels in cancer research. Moreover, such a resource could advance the development of biomarkers based on ion channel signatures for patient stratification and therapy response (Ko et al., 2013).

## Conclusions

Ion channels and transporters are increasingly recognised not merely as regulators of cellular ion homeostasis, but in some cases as active drivers of tumour progression, integrating bioelectrical signals with key oncogenic processes including invasion, hypoxic adaptation, and immune evasion. Despite substantial advances in our understanding of their biological roles, translation into clinical benefit remains limited, in part due to challenges in modelling tumour bioelectrical states and the lack of robust pharmacodynamic markers to monitor ion channel activity *in vivo*. Addressing these gaps will require a shift from descriptive studies towards functional and translational frameworks that link ion flux to clinically measurable outcomes across scales. In this context, the repurposing of ion channel-targeting drugs, particularly those with established safety profiles, offers a promising opportunity to accelerate therapeutic development, especially for suppressing metastatic disease. Integrating advances in electrophysiological tools, imaging technologies, and biologically relevant model systems will be essential to fully exploit the ion transportome in oncology and unlock new avenues for translation and therapeutic intervention.

## Figures and Tables

**Table 1:** Summary of ion channel-targeted therapies (clinical and non-clinical) investigated for the treatment of cancer. ANO1, Anoctamin-1; CRAC, Calcium Release-Activated Calcium; DFS, Disease-Free Survival; EMT, Epithelial-to-Mesenchymal Transition; FOLFOX, Folinic acid, Fluorouracil, and Oxaliplatin; HRQoL, Health-Related Quality of Life; MGMT GB, O^6^-Methylguanine-DNA Methyltransferase promoter methylation in Glioblastoma; NSCLC, Non-Small Cell Lung Cancer; OS, Overall Survival; PFS, Progression-Free Survival; SGLT2, Sodium-Glucose Transporter Type 2; TNBC, Triple-Negative Breast Cancer; TRP, Transient Receptor Potential; VGCC, Voltage-Gated Calcium Channel; VGKC, Voltage-Gated Potassium Channel; VGSC, Voltage-Gated Sodium Channel.

Molecular Target	Therapy	Cancer type	Model System	Effect	Reference
ANO1	Schisandrathera	Prostate and oral cancers	*in vitro* PC-3 and CAL-27 cells	Reduces cell viability through apoptosis	(Park et al., 2023)
ANO1	Zafirlukast	Lung Cancer	*in vitro* LA795 and 16HBE cells	Suppresses metastasis	(Shi et al., 2022)
Ca_v_1.3	Nifedipine	Androgen-sensitive prostate cancer	*in vitro* RWPE-1, LNCaP, PC-3, C4-2, C4,2B and DU145 cells	Decreases cancer cell proliferation	(McKerr et al., 2023)
CRAC	RP4010	Pancreatic ductal adenocarcinoma	*in vitro* MiaPaCa-2, BxPC-3 and L3.6pl cells *in vivo* Patient-derived xenograft mouse model	Suppresses tumor cell proliferation and improves response to chemotherapy	(Khan et al., 2020)
Kir6/SUR	Minoxidil	Gynecological cancers	*in vitro* OVCAR-4, OVCAR-8 and SPEC-2 cells *in vivo* NOD-scid-IL2Rγnull mouse model derived from OVCAR-8 cells	Induces caspase-3 independent cell death	(Fukushiro-Lopes et al., 2020)
K_v_1.3	Memantine	Acute lymphocytic leukaemia cells	*in vitro* Jurkat (JE6-1), F9, JMR, A3, C8 (I.9.2), CEM, HL-60, Molm-13, OCI-AM cellsL, primary cells from healthy donors and primary leukemic cells	Inhibits metastasis and proliferation	(Lowinus et al., 2019)
K_v_10.1	Astemizole	TNBC	*in vitro* MDA-MB-231 cells	Inhibits cell migration	(Valdés-Abadía et al., 2019)
K_v_10.1	Liensinine	Hepatoma	*in vitro* HEK293, HepG2, Huh-7, PC3 and Lo2 cells *in vivo* BALB/c mouse model derived from HepG2 cells	Suppresses tumour cell proliferation and migration	(Ma et al., 2024)
K_v_10.1	Procyanidin Bl	Hepatoma	*in vitro* HEK293, Lo2, Huh-7 and HepG2 cells *in vivo* BALB/c mouse model derived from HepG2 cells	Suppresses tumour cell growth, proliferation and migration	(Na et al., 2020)
Na_v_l.5	E3ab	Ovarian cancer	*in vitro* Caov-3 and MDA-MB-231 cells *in vivo* Nude mouse model derived from Caov-3 cells	Reduces tumour growth and invasion	(Gao et al., 2019)
TRPC6	BI-749327	Breast cancer	*in vitro* MDA-MB-231,MDA-MB-231-TE3, CAL-51, HCC-1806 and HMLER cells Breast cancer-derived organoids *in vivo* Patient-derived xenograft mouse model	Inhibits mammosphere formation	(Mukhopadhyay et al., 2023)
TRPV1	AMG9810	Cervical cancer	*in vitro* CaSki, HEK293, HeLa, H1299, SiHa, SNU719, AGS, SNU668, MKN28 and YCC2 cells *in vivo* NOD/SCID mouse model derived from CaSki CR cells	Reverses cisplatin resistance	(Oh et al., 2023)
TRPV6	mAb82	Prostate cancer	*in vitro* PC3M, LNCaP and HEK293 cells *in vivo* Swiss nude mouse model derived from PC3M cells	Decreases prostate cancer cell survival and reduced tumour growth	(Haustrate et al., 2024)
T-type VGCC	KTt-45	Various cancer cells	*in vitro* A549, HeLa, MCF-7, Raji and BJ-5ta cells	Induces apoptosis	(Du et al., 2023)
VGCCs	Benidipine, Lacidipine, Lomerizine, Manidipine and Nifedipine	Ovarian cancer	*in vitro* A2780 and A27800-SP cells *in vivo* BALB/c-nu/nu mouse model derived from A2780-AD and A2780-SP cells	Reduces expression of cancer stem cell markers	(Lee et al., 2020)
VGSCs	JZTX-14	TNBC	*in vitro* MDA-MB-231 cells	Impairs EMT	(Wu et al., 2023)
Kir6/SUR	Minoxidil	Gynecological cancers	Clinical study Phase II NCT05272462	To be determined (Trial recruiting)	-
L-type VGCC	Amlodipine	Endometrial cancer	Clinical study Phase II NCT06942377	To be determined (Trial recruiting)	-
SERCA	Mipsagargin	Advanced solid tumours	Clinical study Phase I NCT01056029	Prolongs disease stabilisation for a subset of patients	(Mahalingam et al., 2016)
SERCA	Mipsagargin	Progressive advanced Hepatocellular carcinoma	Clinical study Phase II NCT01777594	Prolongs disease stabilisation for patients	(Mahalingam et al., 2019)
SGLT2	Dapagliflozin	Breast cancer	Clinical Study Phase II NCT06341842	To be determined (Trial recruiting)	(Greco et al., 2025)
TRPV6	Soricidin-13	Advanced solid tumours	Clinical study Phase I NCT01578564	Evidence of stable disease	(Fu et al., 2017)
VGCCs	Carboxyamidotriazole	Advanced NSCLC	Clinical study Phase III CTR20160395	Increases PFS	(Si et al., 2020)
VGCCs	Carboxyamidotriazole	Glioblastoma and anaplastic gliomas	Clinical study Phase Ib NCT01107522	Improved PFS and OS compared to standard chemotherapy approach	(Omuro et al., 2018)
VGKCs	Fampridine in combination with imatinib	KIT mutant gastrointestinal stromal tumours	Clinical study Phase I NCT07171203	To be determined (Trial recruiting)	-
VGSCs	Lidocaine	Breast cancer	Clinical study Phase III NCT01916317	Increases DFS and OS	(Badwe et al., 2023)
VGSCs	Lidocaine	Colorectal cancer	Clinical study Pilot NCT02786329	Decreases rate of recurrence	(Alexa et al., 2023)
VGSCs and glutamate release	Riluzole	Metastatic colorectal cancer	Clinical Study Phase I NCT04761614	Improves treatment response	(Li et al., 2025)
VGSCs and some ion transporters	Cannabinoids	MGMT Glioblastoma	Clinical study Phase II NCT05629702	To be determined (Trial recruiting)	(Bhaskaran et al., 2024)

## Glossary

Ca^2+^: Calcium
CaM: Ca^2+^/calmodulin
Cl^-^: Chloride
ECM: Extracellular Matrix
EMT: Epithelial-to-Mesenchymal Transition
H^+^: Hydrogen
HCC: Hepatocellular carcinoma
HIF: Hypoxia-Inducible Factor
K^+^: Potassium
Na^+^: Sodium
NCX: Na^+^/Ca^2+^ exchanger
NKA: Na^+^/K^+^ ATPase
O_2_: Oxygen
SGLT2: Sodium-Glucose Cotransporter 2
TNBC: Triple-Negative Breast Cancer
VGCC: Voltage-Gated Ca^2+^ Channel
VGKC: Voltage-Gated K^+^ Channel
VGSC: Voltage-Gated Na^+^ Channel
V_m_: Membrane Potential
